# Neuroprotective Effects of Pharmacological Hypothermia on Hyperglycolysis and Gluconeogenesis in Rats after Ischemic Stroke

**DOI:** 10.3390/biom12060851

**Published:** 2022-06-19

**Authors:** Longfei Guan, Hangil Lee, Xiaokun Geng, Fengwu Li, Jiamei Shen, Yu Ji, Changya Peng, Huishan Du, Yuchuan Ding

**Affiliations:** 1 China-America Institute of Neuroscience, Beijing Luhe Hospital, Capital Medical University, Beijing 101149, China; guanlongfei1989@163.com (L.G.); fengwulijlu@126.com (F.L.); shenjiameide@163.com (J.S.); dhs139106@126.com (H.D.); 2 Department of Neurosurgery, Wayne State University School of Medicine, Detroit, MI 48201, USA; hangil.lee@med.wayne.edu (H.L.); doc.jiyu@foxmail.com (Y.J.); cpeng@med.wayne.edu (C.P.); 3 Department of Neurology, Beijing Luhe Hospital, Capital Medical University, Beijing 101149, China; 4 Department of General Surgery, Beijing Luhe Hospital, Capital Medical University, Beijing 101149, China

**Keywords:** stroke, ischemia/reperfusion injury, combination therapy of chlorpromazine and promethazine (C + P) with dihydrocapsaicin (DHC), glycolysis, gluconeogenesis

## Abstract

Stroke is a leading threat to human life. Metabolic dysfunction of glucose may play a key role in stroke pathophysiology. Pharmacological hypothermia (PH) is a potential neuroprotective strategy for stroke, in which the temperature is decreased safely. The present study determined whether neuroprotective PH with chlorpromazine and promethazine (C + P), plus dihydrocapsaicin (DHC) improved glucose metabolism in acute ischemic stroke. A total of 208 adult male Sprague Dawley rats were randomly divided into the following groups: sham, stroke, and stroke with various treatments including C + P, DHC, C + P + DHC, phloretin (glucose transporter (GLUT)-1 inhibitor), cytochalasin B (GLUT-3 inhibitor), TZD (thiazolidinedione, phosphoenolpyruvate carboxykinase (PCK) inhibitor), and apocynin (nicotinamide adenine dinucleotide phosphate oxidase (NOX) inhibitor). Stroke was induced by middle cerebral artery occlusion (MCAO) for 2 h followed by 6 or 24 h of reperfusion. Rectal temperature was monitored before, during, and after PH. Infarct volume and neurological deficits were measured to assess the neuroprotective effects. Reactive oxygen species (ROS), NOX activity, lactate, apoptotic cell death, glucose, and ATP levels were measured. Protein expression of GLUT-1, GLUT-3, phosphofructokinase (PFK), lactate dehydrogenase (LDH), PCK1, PCK2, and NOX subunit gp91 was measured with Western blotting. PH with a combination of C + P and DHC induced faster, longer, and deeper hypothermia, as compared to each alone. PH significantly improved every measured outcome as compared to stroke and monotherapy. PH reduced brain infarction, neurological deficits, protein levels of glycolytic enzymes (GLUT-1, GLUT-3, PFK and LDH), gluconeogenic enzymes (PCK1 and PCK2), NOX activity and its subunit gp91, ROS, apoptotic cell death, glucose, and lactate, while raising ATP levels. In conclusion, stroke impaired glucose metabolism by enhancing hyperglycolysis and gluconeogenesis, which led to ischemic injury, all of which were reversed by PH induced by a combination of C + P and DHC.

## 1. Introduction

Stroke is one of the leading causes of death and disability worldwide [1,2]. Among them, ischemic stroke caused by an embolic or thrombotic occlusion of a cerebral artery accounts for the majority [3]. Recanalization and reperfusion are the mainstays of acute stroke treatment and can reduce infarct sizes and reverse neurologic deficits [4,5]. Current clinical management focuses on rapid reperfusion with intravenous thrombolysis and endovascular thrombectomy, which benefits only a small proportion of stroke patients due to the narrow therapeutic window and contraindications [6,7]. Despite increasing rates of recanalization, 50% of the patients still end up with disabilities after ischemic events [8]. Therefore, identification of new therapeutic modalities is critical to improve stroke outcomes. As the STAIR conferences recommended, there are ample opportunities to study and develop new neuroprotective agents, as well as repurposing known agents, as adjunct treatments to reperfusion therapy in the era of highly effective reperfusion [9,10].

Therapeutic hypothermia (TH) has been recognized as one of the most effective potential treatments of stroke [11,12,13]. TH decreases metabolic activity, reactive oxygen species (ROS), and inflammation [14], thus preventing the expansion of irreversible injury and saving reversible ischemic penumbra. However, delayed induction of cerebral hypothermia, increased incidence of pneumonia, and intensive labor limit its application in the clinical setting [15]. Pharmacological hypothermia (PH) is an alternative to physically-induced hypothermia, in which the temperature is carefully decreased by drugs without the risks and labor of traditional hypothermia [16]. Dihydrocapsaicin (DHC) and two phenothiazines, chlorpromazine and promethazine (C + P), are a combination that has proven to be efficacious in animal studies [17]. DHC is a capsaicin analog that activates the transient receptor potential vanilloid 1 (TRPV1) receptor, the finding of which was awarded the 2021 Nobel Prize in physiology and medicine [18], and confers hypothermia by decreasing metabolism [19]. C + P are neuroleptic drugs that induce an “artificial hibernation”-like state [20].

To date, it has been observed that increased ROS levels, glycolysis, and lactic acidosis are responsible for brain tissue injury and death [21]. However, therapies that specifically target these domains did not ameliorate ischemic damage [22]. One of the candidates of the missing link may be gluconeogenesis. Recent studies found that the brain also undergoes gluconeogenesis, which is usually overshadowed by the organs we typically associate with generating glucose de novo such as the liver, intestines, kidneys, and muscle [23]. When the brain undergoes acute ischemic stroke (AIS), gluconeogenesis in the brain tissue is interrupted due to a shortage of ATP, leading to the accumulation of undesirable side products. These side products, which include lactic acid and ROS, contribute to cellular injury and death [24]. The lack of consideration for gluconeogenesis may have caused the failure of previous therapies, which targeted downstream side products rather than their source. Hyperglycolysis may be yet another contributor to brain injury that deserves greater attention. It has been observed to correlate with greater brain injury [25], possibly through mitochondrial oxidative phosphorylation decoupling [26]. Specifically in AIS, hyperglycolysis contributes to the energy crisis that ultimately leads to brain damage [27]. More convincingly, many treatment modalities have ameliorated AIS damage by decreasing hyperglycolysis [28,29], one of which includes C + P [30].

Although it has been shown that C + P + DHC offers neuroprotection [17], there is much that remains to be studied. While it is clear that the combination is efficacious, the underlying molecular mechanism of efficacy is yet to be elucidated. More evidence is needed to show that the combination better enhances neuroprotection, as compared to each formulary alone. Whether glucose metabolism plays a role in amelioration of AIS by C + P + DHC also remains to be explored. Finally, although C + P + DHC has been described to ameliorate brain damage, its dependence or independence to hypothermia requires further validation [24,30].

The present study determined whether PH with C + P and/or DHC led to neuroprotection by improving glucose metabolism in acute ischemic stroke. More specifically, we hypothesized that the induction of hyperglycolysis and gluconeogenesis, and thus their contribution to oxidative injury and acidosis in ischemia/reperfusion injury, can be reduced by PH. The conclusions we draw may unlock new treatment modalities that better prevent brain tissue death in stroke.

## 2. Results

### 2.1. Physiological Parameters

Physiological parameters were measured before surgery (Pre MCAO), after 2 h MCA occlusion (pre reperfusion), and after 2 h reperfusion. A catheter was inserted into the right femoral artery for continuous monitoring of mean arterial pressure (MAP) and periodic analyses of blood gases and pH [24,31]. There were no significant differences in blood pH, pO_2_, pCO_2_, or mean arterial pressure (MAP) between the groups (data are shown in the supplemental materials).

### 2.2. Induction of Pharmacological Hypothermia

**Figure 1:**
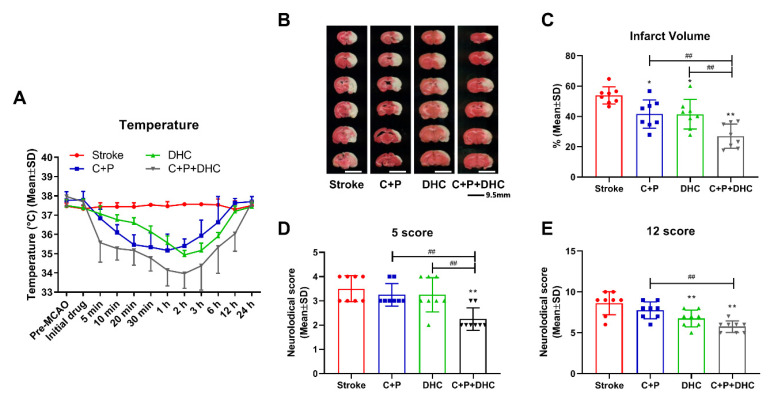
Induction of pharmacological hypothermia and its neuroprotective effects in rat MCAO models. (**A**) Body temperature before and after the model development with different treatments. Body temperature reached the lowest target of 35.2 °C by C + P, 34.9 °C by DHC, and 34 °C by C + P + DHC. (**B**) Representative brain slices stained with TTC after 2 h MCAO and 48 h of reperfusion. (**C**) Graphic quantification of TTC sections revealing that C + P + DHC result in significantly decreased infarct volume. Neurological deficits after 2 h MCAO and C + P, DHC, or C + P + DHC therapy using the 5 score system (**D**) and 12 score system (**E**) C + P + DHC combination therapy significantly reduced neurological deficits. Data are presented as mean ± SD, * *p* < 0.05, ** *p* < 0.01, as compared to stroke group; ## *p* < 0.01, as compared to C + P or DHC alone (*n* = 8).

By using a circulating heating pad, body temperature remained at 37.4 °C in the stroke group without treatment, while other groups receiving different treatments (C + P, DHC, and C + P + DHC) had their body temperature significantly reduced (Figure 1A). From reperfusion onset, C + P and/or DHC reduced temperatures to hypothermic levels (below 36 °C) from 20 min to 1 h. Combination of C + P and DHC induced a faster (at 5 min), longer (up to 6 h of reperfusion), and deeper (low temperature at 34 °C) hypothermia. Eventually the body temperatures spontaneously returned to normal levels.

### 2.3. Infarct Volume and Neurological Deficits

An infarct volume of 53.9% was obtained at 48 h reperfusion after 2 h MCAO. Infarctions were reduced to 41.6% and 41.5% by C + P and DHC respectively (*p* < 0.05). With the combination of C + P and DHC, infarct volume was significantly further reduced to 27.0%, as compared to untreated stroke (*p* < 0.01) and the single treatment groups (*p* < 0.01) (Figure 1B,C). Neurological deficits in the 2 h MCAO group followed by 48 h of reperfusion were determined by the 5 or 12 score systems. Similarly, the combination of C + P and DHC significantly reduced neurological deficits when compared to the untreated (*p* < 0.01) or single treatment groups (*p* < 0.01). C + P alone did not show significant improvement in neurological deficits, while DHC showed an improvement in neurological deficits with only the 12 score scale (*p* < 0.01) (Figure 1D,E).

### 2.4. ROS Levels

**Figure 2:**
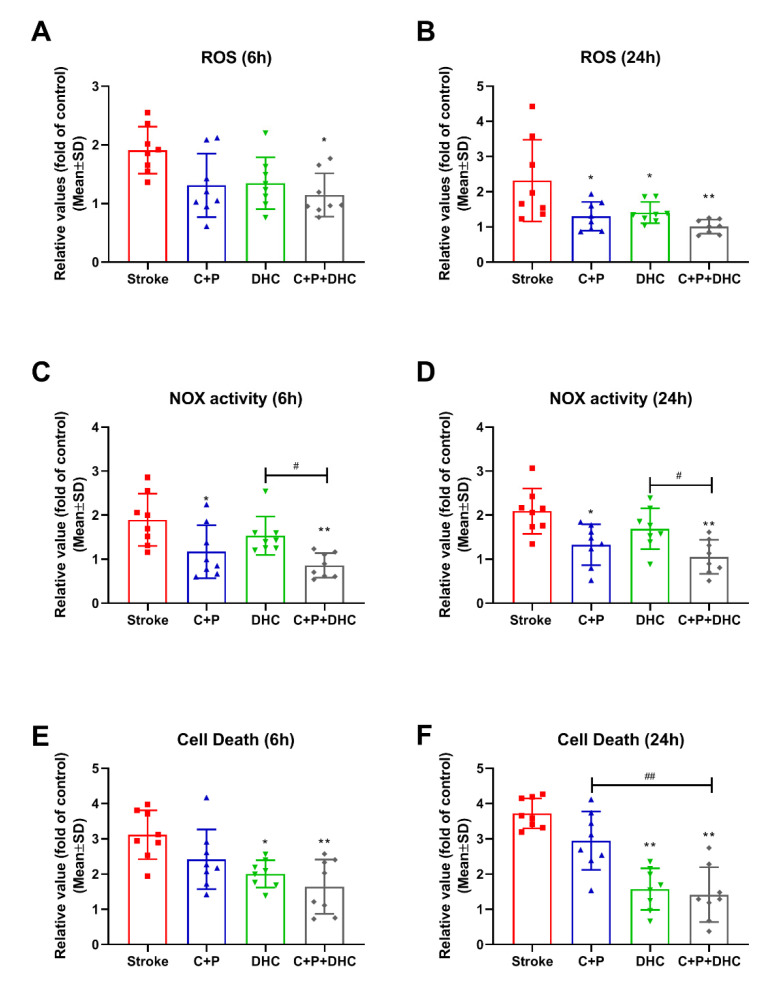
Effect of pharmacological hypothermia on ROS, NOX activity, and cell death in rats with 2 h MCAO. Stroke increased ROS levels, which was significantly reduced by the combination of C + P and DHC at both 6 (**A**) and 24 h (**B**) after reperfusion. C + P and DHC individually significantly reduced ROS levels at 24 h after reperfusion (**B**). NOX activity was significantly increased after stroke at 6 (**C**) and 24 h (**D**) of reperfusion, which was reversed by C + P, or the combination of C + P and DHC treatment. Apoptotic cell death significantly increased at 6 (**E**) and 24 h (**F**) of reperfusion. DHC and the combination of C + P and DHC significantly reduced cell death, while C + P alone did not. Data are presented as mean ± SD, * *p* < 0.05, ** *p* < 0.01, as compared to stroke group; # *p* < 0.05, ## *p* < 0.01, as compared to C + P or DHC alone (*n* = 8).

Stroke induced a significant increase in ROS production at 6 and 24 h reperfusion compared to the sham-operated group (reference as 1). C + P or DHC significantly reduced ROS levels at 24 h after reperfusion, but no significant reductions were observed at 6 h. The combination of C + P and DHC significantly reduced ROS level at both 6 (*p* < 0.05) and 24 h (*p* < 0.01) after reperfusion (Figure 2A,B).

### 2.5. NOX Activity

Similarly, a significantly elevation in NOX activity was observed at 6 and 24 h of reperfusion compared to the sham-operated group (reference as 1), which was reversed by C + P treatment (*p* < 0.05 for 6 h; *p* < 0.01 for 24 h) and the combination treatment (*p* < 0.01 for both groups). DHC, however, did not reduce NOX activity (Figure 2C,D).

### 2.6. Apoptotic Cell Death

Apoptotic cell death was greatly increased in the stroke group compared to the sham-operated group (reference as 1). DHC decreased cell death at 6 (*p* < 0.05) and 24 h (*p* < 0.01) of reperfusion, while C + P alone did not. Again, combination of C + P and DHC further decreased cell death (*p* < 0.01 for 6 and 24 h) (Figure 2E,F).

### 2.7. Lactate Level

**Figure 3:**
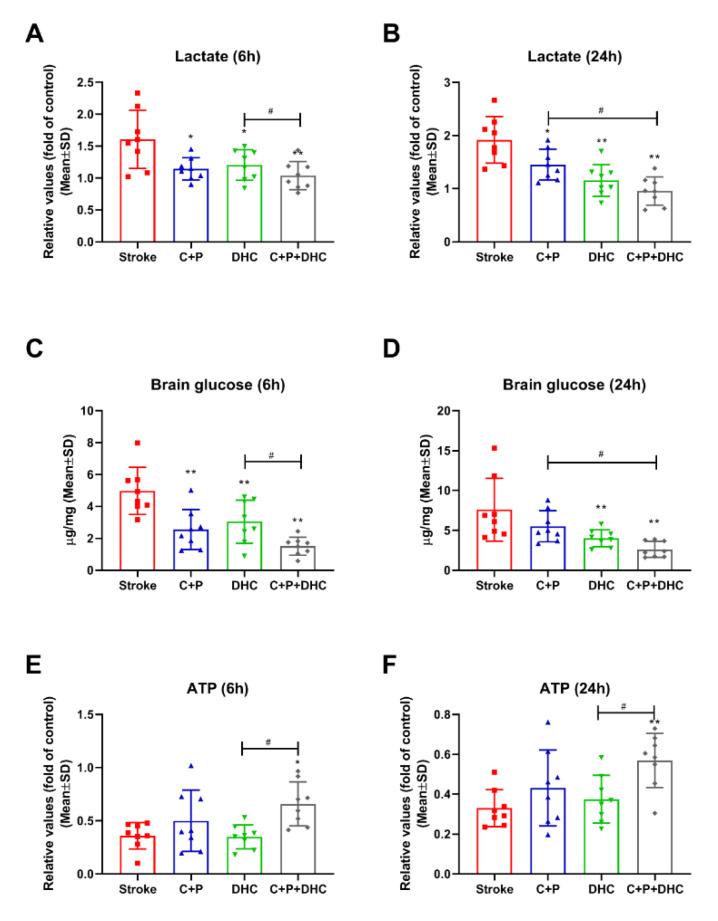
Effect of pharmacological hypothermia on lactate, glucose, and ATP levels in rats with 2 h MCAO. Lactate level was significantly increased after stroke, which was significantly reduced by C + P, DHC, or combination treatment at both 6 (**A**) and 24 h (**B**) after reperfusion. Brain glucose levels increased at 6 (**C**) and 24 h (**D**) of reperfusion. DHC or the combination of C + P and DHC decreased brain glucose level at both 6 and 24 h of reperfusion, while C + P decreased brain glucose level at 6 h of reperfusion. Stroke reduced ATP levels, and only the combination therapy significantly increased ATP levels at both 6 (**E**) and 24 h (**F**) after reperfusion. Data are presented as mean ± SD, * *p* < 0.05, ** *p* < 0.01, as compared to stroke group; # *p* < 0.05, as compared to C + P or DHC alone (*n* = 8).

Compared to the sham-operated group (reference as 1), lactate was significantly increased after stroke. Both C + P and DHC alone decreased lactate levels at 6 (*p* < 0.05 for C + P and DHC) and 24 h (*p* < 0.05 for C + P; *p* < 0.01 for DHC) of reperfusion. Again, the combination treatment enhanced lactate reduction at 6 and 24 h (*p* < 0.01), as compared to C + P alone at 24 h (*p* < 0.05) and DHC alone at 6 h (*p* < 0.05) of reperfusion. (Figure 3A,B).

### 2.8. Cerebral Glucose Concentration

A significant increase in cerebral glucose level was observed after stroke as compared to the sham-operated group, suggesting hyperglycemia. DHC decreased cerebral glucose levels at both 6 (*p* < 0.01) and 24 h (*p* < 0.01) reperfusion, while C + P decreased cerebral glucose levels at 6 h (*p* < 0.01). The combination treatment greatly decreased cerebral glucose levels at 6 and 24 h (*p* < 0.01) as compared to C + P alone at 24 h (*p* < 0.05) and DHC alone at 6 h (*p* < 0.01) (Figure 3C,D).

### 2.9. ATP Level

ATP level was greatly decreased in the stroke group compared to the sham-operated group (reference as 1). The combination of C + P and DHC further prevented ATP depletion at both 6 (*p* < 0.05) and 24 h (*p* < 0.01) (Figure 3E,F).

### 2.10. Hypothermia Induced Neuroprotection Is Associated with Glucose Metabolism

**Figure 4:**
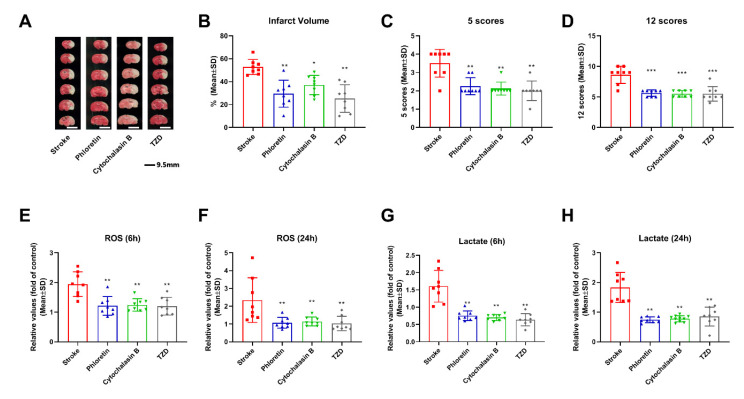
Effects of GLUT-1, GLUT-3, and PCK inhibitors’ neuroprotection in ischemic stroke rats. (**A**) Representative brain slices stained with TTC after 2 h MCAO and 48 h of reperfusion. (**B**) Infarct volumes quantified with TTC sections showing that phloretin, cytochalasin B, and TZD significantly decreased infarct volumes. Phloretin, cytochalasin B, and TZD significantly reduced neurological deficits, as evaluated by the 5 (**C**) and 12 score (**D**) scales. ROS levels were significantly decreased by phloretin, cytochalasin B, and TZD at 6 (**E**) and 24 h (**F**) of reperfusion. Phloretin, cytochalasin B, and TZD significantly decreased lactate levels at both 6 (**G**) and 24 h (**H**) after reperfusion. Data are presented as mean ± SD, * *p* < 0.05, ** *p* < 0.01, *** *p* < 0.001, as compared to stroke group (*n* = 8).

To further elucidate whether the neuroprotective mechanism is related to glucose metabolism after ischemia stroke, the inhibitors of GLUT-1 (phloretin), GLUT-3 (cytochalasin B), and PCK (TZD) were used. As shown in Figure 4, phloretin, cytochalasin B, and TZD significantly decreased the infarct volumes to 29.4%, 37.1%, and 25.2% respectively (Figure 4A,B). Similarly, all three inhibitors significantly reduced neurological deficits as measured by the 5 and 12 score scales (*p* < 0.01) (Figure 4C,D). ROS levels were also significantly reduced by phloretin, cytochalasin B, and TZD at 6 (*p* < 0.01) and 24 h (*p* < 0.01) of reperfusion (Figure 4E,F). In addition, phloretin, cytochalasin B, and TZD significantly decreased lactate levels at both 6 (*p* < 0.01) and 24 h (*p* < 0.01) of reperfusion (Figure 4G,H).

### 2.11. Pharmacological Hypothermia Reduced Expression of Glycolytic Enzyme

**Figure 5:**
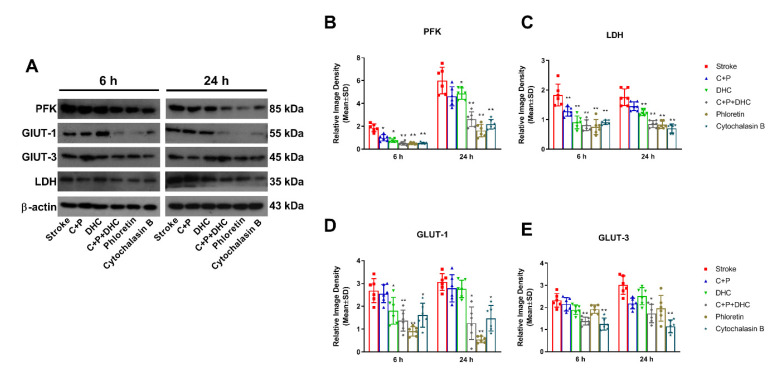
Effect of pharmacological hypothermia on glycolytic enzymes. (**A**) Representative Western blot bands of glycolytic enzymes. PFK (**B**), LDH (**C**), GLUT-1 (**D**), and GLUT-3 (**E**) levels were significantly reduced by pharmacological hypothermia, phloretin, and cytochalasin B. Data are presented as mean ± SD, * *p* < 0.05, ** *p* < 0.01, as compared to stroke group (*n* = 6).

Ischemic rats with 2 h MCAO exhibited a significant increase in protein levels of PFK, LDH, GLUT-1, and GLUT-3 at 6 and 24 h of reperfusion, as compared to the sham group (referenced as 1) (Figure 5A–I). PFK increase was significantly diminished by C + P alone at 6 h (*p* < 0.05) and DHC alone at 6 and 24 h (*p* < 0.05), while their combination enhanced the reduction at both 6 (*p* < 0.01) and 24 h (*p* < 0.01). Similar patterns were achieved by phloretin and cytochalasin B at both 6 (*p* < 0.01) and 24 h (*p* < 0.01) of reperfusion. Again, LDH levels were significantly decreased by C + P at 6 h (*p* < 0.01) and DHC at 6 (*p* < 0.01) and 24 h (*p* < 0.01), with further enhancement by the combination of C + P and DHC, phloretin, and cytochalasin B at 6 and 24 h (*p* < 0.01) (Figure 5B–E). The same trends were observed in GLUT-1 and GLUT-3 protein levels. The combination of C + P and DHC significantly decreased GLUT-1 and GLUT-3 at both 6 (*p* < 0.01) and 24 h (*p* < 0.05) of reperfusion, while DHC only decreased GLUT-1 expression at 6 h of reperfusion (*p* < 0.05). Phloretin inhibited the expression of GLUT-1 significantly at 6 (*p* < 0.01) and 24 h (*p* < 0.05), while cytochalasin B inhibited the expression of GLUT-1 (*p* < 0.05) and GLUT-3 (*p* < 0.01) at 6 and 24 h of reperfusion (Figure 5F–I).

### 2.12. Pharmacological Hypothermia Reduced Expression of Gluconeogenic Enzymes

**Figure 6:**
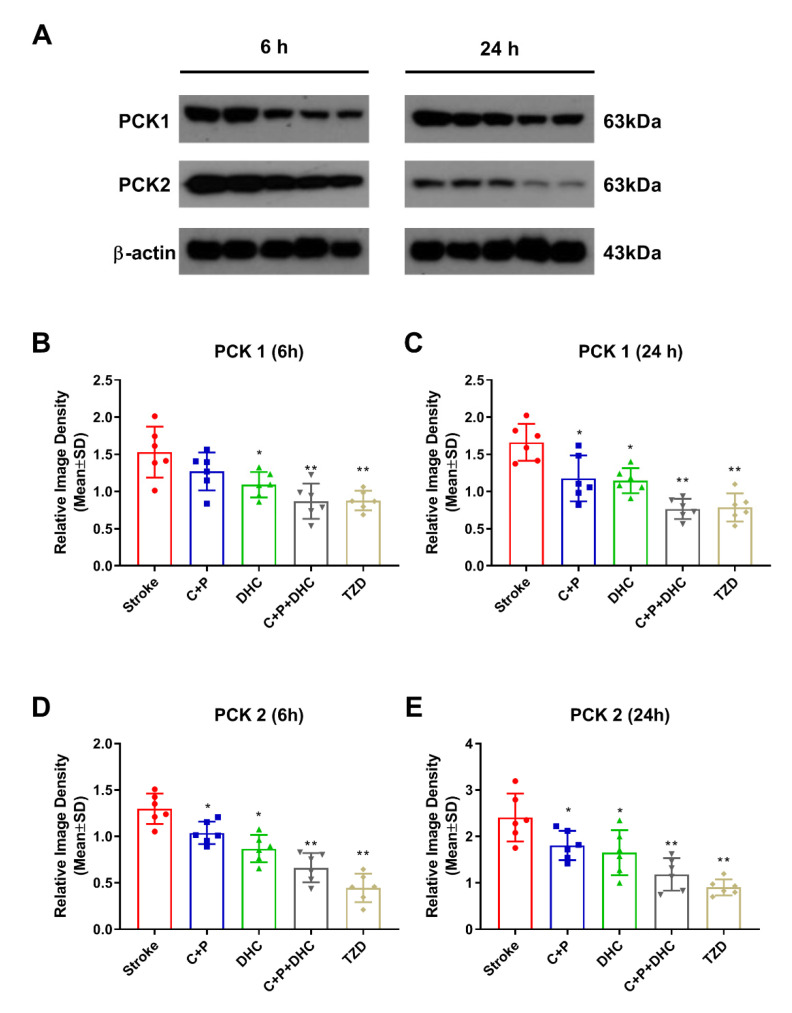
Effect of pharmacological hypothermia on gluconeogenic enzymes. (**A**) Representative Western blot bands of gluconeogenic enzymes. PCK1 (**B**,**C**) and PCK2 (**D**,**E**) levels were significantly reduced by pharmacological hypothermia and TZD. Data are presented as mean ± SD, * *p* < 0.05, ** *p* < 0.01, as compared to stroke group (*n* = 5).

Ischemic rats with 2 h MCAO significantly increased PCK1 and PCK2 protein levels at 6 and 24 h of reperfusion, as compared to the sham group (referenced as 1) (Figure 6A–E). The increase in PCK1 was significantly reduced by C + P at 24 h (*p* < 0.05) and DHC at 6 and 24 h (*p* < 0.05). Their combination provided stronger inhibition at both 6 and 24 h (*p* < 0.01) of reperfusion. The inhibitive effect was similarly induced by TZD, the PCK inhibitor (*p* < 0.01) (Figure 6B,C). The increase in PCK2 after stroke was significantly reduced by all of the PH protocols at both time points, similar to the effects induced by TZD (*p* < 0.01) (Figure 6D,E).

### 2.13. Effect of NOX on the Neuroprotection Induced by Pharmacological Hypothermia

**Figure 7:**
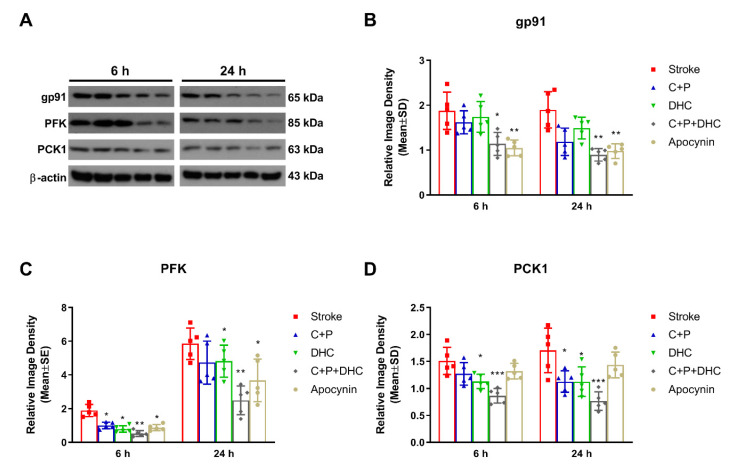
Effect of NOX inhibition on glucose metabolism enzymes. (**A**) Representative Western blot bands of gp91, PFK, and PCK1. gp91 (**B**), PCK1 (**C**) and PFK (**D**) levels were significantly reduced by pharmacological hypothermia. Apocynin reduced gp91 and PFK protein expressions, while PCK1 protein expression was not reduced. Data are presented as mean ± SD, * *p* < 0.05, ** *p* < 0.01, *** *p* < 0.001, as compared to stroke group (*n* = 5).

To further explore whether PH-induced neuroprotection was associated with NOX, apocynin (a NOX inhibitor) was applied. The combination of C + P and DHC significantly decreased the protein level of gp91 at 6 (*p* < 0.05) and 24 h (*p* < 0.01) of reperfusion, which was consistent with the effect induced by apocynin (*p* < 0.01) (Figure 7A–C). The key glycolytic enzyme PFK was significantly reduced by C + P at 6 h (*p* < 0.05), DHC at 6 and 24 h (*p* < 0.05), and the combination of C + P and DHC at both 6 (*p* < 0.01) and 24 h (*p* < 0.01). These were similar to the reductions induced by apocynin at both 6 and 24 h (*p* < 0.05) of reperfusion (Figure 7D,E). In addition, while the key gluconeogenic enzyme PCK1 was reduced by PH, it was not reduced by apocynin, suggesting that PCK1 was independent from NOX activity (Figure 7F,G).

## 3. Discussion

The present study revealed that the combination of C + P (4 mg/kg) and DHC (0.5 mg/kg) induced an effective hypothermic state, and that the combination induced enhanced neuroprotection as compared to each alone. This neuroprotection was evidenced by a reduction in infarct volumes, neurological deficits, NOX activity and its subunit gp91, ROS, cell death, glucose, and lactate, with simultaneous increase in ATP levels and improved overall glucose metabolism, with consideration for both hyperglycolysis and gluconeogenesis. The improvement of glucose metabolism with C + P + DHC was evidenced by decreased levels of glycolytic enzymes (GLUT-1, GLUT-3, PFK and LDH) and inhibition of NOX and gluconeogenic enzymes (PCK1 and PCK2). These findings support our hypothesis that PH, especially induced by the combination of C + P and DHC, reversed dysfunctional hyperglycolysis and gluconeogenesis induced by stroke, resulting in reduced brain damage.

Systemic hypothermia is a traditional therapeutic hypothermia method to prevent brain cell death from acute ischemic stroke. Early attempts at harnessing hypothermia to prevent brain tissue death from acute ischemic stroke frequently utilized physical means of decreasing the systemic temperature. Clinical limitations of physical hypothermia induction included delays in cooling initiation and the onset of the target temperature. The late start necessitated a prolonged hypothermia duration, which, in turn, required intensive medical support and caused secondary complications such as pneumonia [12]. The only means of physically inducing hypothermia that found significant success was selective hypothermia (or regional cooling), achieved through injecting cool saline in the midst of a mechanical thrombectomy procedure [32,33]. While highly effective, this procedure has a stringent inclusion criteria, limiting the patient population that it benefits. Pharmacologic cooling is attractive because of its ease of use. Although it induces systemic hypothermia, pharmacological hypothermia (PH) is a strong stroke therapy candidate that may be able to provide the benefits of cooling without the intense labor, unintended consequences, and limited patient population of physical hypothermia. Furthermore, it also counteracts the physiological resistance to cooling by inhibiting shivering [16,34]. While single drugs require toxic doses to achieve therapeutic levels of hypothermia, combinations of lower doses avoid toxicity and work synergistically to achieve efficient cooling [17]. There are eight classes of pharmacological agents that can induce hypothermia, which are the cannabinoid system, the transient receptor potential vanilloid channel 1 (TRPV1) receptor, the opioid receptor, neurotensin, thyroxine derivatives, dopamine receptor activators, gaseous hypothermia, and adenosine/adenine nucleotides.

DHC, an analog and congener of capsaicin in chili peppers (capsicum), is a TRPV1 agonist [18,35]. TRPV1 is a nonspecific cation channel and confers neuroprotection through its ability to induce hypothermia [34]. Activation of TRPV1 resets thermoregulation into the hypothermic range, which permits effective physiological hypothermia, while eliminating concerns for natural rewarming responses such as shivering [36]. The thermoregulation reset is achieved in several brain regions, including the preoptic area of the hypothalamus [37]. However, given the toxicity and complications associated with high doses of DHC that are required to achieve effective hypothermia, its use as monotherapy is limited [38,39]. Previously, we have demonstrated that a low dose (0.5 mg/kg) DHC combined with physical hypothermia (ice pad) could provide enhanced hypothermia and neuroprotection [39]. Additionally, DHC was seen to act synergistically at low, non-toxic doses with phenothiazine-class drugs to induce effective PH [17].

Chlorpromazine and promethazine (C + P), two members of the phenothiazine class of neuroleptic drugs, have been widely used for their antipsychotic and sedative effects [40]. Previously, we have reported that C + P could confer neuroprotection in stroke via the induction of an “artificial hibernation”-like state, achieved by decreasing brain activity and glucose metabolism [41]. The depressive effect on glucose utilization is similar to that of local anesthetics [42]. Combining physical hypothermia with C + P significantly enhanced the neuroprotective effects of mild hypothermia [43]. Most importantly, C + P, as members of the phenothiazine class, act synergistically with DHC, inducing hypothermia without concern for toxic doses.

The mechanism of ischemic damage has been well described. In sufficiently perfused brain tissue, ATP is primarily generated by oxidative phosphorylation in the mitochondria [44]. In hypoxic conditions, such as AIS, mitochondrial oxidative phosphorylation is no longer possible. Instead, the ischemic brain tissue depends on glycolysis, a considerably inefficient method compared to the perfused state [45]. Thus, the brain attempts to compensate with excessive glucose uptake through GLUT-1 and GLUT-3 transporters, leading to neuronal injury [46]. This state of increased glucose uptake and metabolism is described as hyperglycemia and hyperglycolysis, which is frequently seen in ischemia/reperfusion injuries and associated with poor outcomes [29,30,47]. Pharmacological and physical hypothermia have been seen to target this pathway effectively. Pharmacological hypothermia with DHC and C + P suppressed ROS and lactic acid accumulation and prevented ATP depletion in achieving neuroprotection [17].

In the present study, hyperglycolysis inhibition attenuated brain damage, as observed by the relationship between improved amelioration and decreased glucose, lactate, and PFK. Recent studies indicate that hyperglycolysis-exacerbated injury, especially during reperfusion, is due to the activation of NOX, independent of lactic acidosis [48]. NOX is a multi-component (p47^phox^, p67^phox^, p40^phox^ and Rac2) membrane-bound enzyme complex located in both the cytosol and plasma membrane [49]. When NOX is phosphorylated at its p47^phox^ subunit, it forms a complex and translocates to the plasma membrane to dock with specific plasma membrane subunits such as gp91^phox^ [50]. The catalytic core of the enzyme is composed of gp91^phox^ [51]. NOX is dependent on glucose metabolism, specifically the hexose monophosphate shunt, which supplies the NADPH necessary for enzymatic activity [52]. NADPH is a necessary cofactor for NOX as it transfers its electron to O_2_ to create superoxide (O_2_^−^) [53]. Hence, the presence of glucose during reperfusion increases neuronal NOX activity [54,55] by functioning as the requisite electron donor for neuronal superoxide production through the generation of NADPH [54]. Moreover, activity of the catalytic subunit, gp91^phox^, is dependent on the presence of NADPH produced by glycolysis [54]. After ischemia/reperfusion injury, there may be a time window in which hyperglycolysis-induced NOX activation enhances ROS generation. The present study found that stroke exacerbates NOX activation, ROS generation, and hyperglycolysis, which were reduced by C + P and DHC, suggesting that PH induced neuroprotection via improved glucose metabolism with NOX inhibition.

Inhibiting hyperglycolysis has proven to be therapeutic in contexts other than AIS. In peritoneal dialysis, peritoneal fibrosis is prevented through hyperglycolysis inhibition [56]. In thromboembolic cerebral ischemia, neuroprotection was induced with ethanol and therapeutic hypothermia through hyperglycolysis attenuation [57]. In AIS, various ameliorative therapies were observed to confer benefit while inhibiting hyperglycolysis. Ethanol and modafinil, each of which are known to be neuroprotective, inhibited hyperglycolysis in attenuating AIS damage in combination [28]. Normobaric O_2_ [29] and ischemic pre-conditioning [58] modulated signaling pathways to subdue hyperglycolysis in postischemic states. Most relevant to this study, C + P also attenuated hyperglycolysis [30].

More recently, it has been observed that the brain also undergoes gluconeogenesis. One of the crucial means of maintaining glucose levels in humans is gluconeogenesis. The common perception is that gluconeogenic activities are only present in the liver, kidneys, intestines, and muscle tissue. However, many studies have now proven that the brain also undergoes gluconeogenesis in significant levels. While the organs that are classically associated with gluconeogenesis usually overshadow it, the brain is definitely capable of, and undergoes gluconeogenesis [23]. This may be the missing link to our understanding of the mechanism of damage induced by AIS. In an attempt to compensate for ATP depletion, the ischemic brain tissue initiates gluconeogenesis. However, due to ATP depletion, gluconeogenesis is incomplete and results in the undesirable byproduct of lactic acid instead. The consequence of dysfunctional gluconeogenesis of the ischemic brain tissue is an excessively active phosphoenolpyruvate carboxy kinase (PCK) enzyme, which contributes to the neurotoxic pathways of lactic acid and ROS accumulation [23]. In ischemia, when mitochondrial oxidative phosphorylation and ATP production are disrupted, anaerobic glycolysis becomes the primary source of ATP. Anaerobic glycolysis alone cannot produce sufficient ATP to maintain brain function and produces lactate, leading to acidosis and ROS. In an attempt to compensate for insufficient energy, we see a full circle where gluconeogenesis is increasingly attempted after ischemia to provide additional substrate for energy production, which may not function correctly due to a lack of ATP [23], leading to excess lactic acidosis. We must note that gluconeogenesis is anabolic, which increases the amount of glucose, while oxidative phosphorylation is catabolic and decreases the amount of glucose. In our previous study, cerebral gluconeogenesis was actively found after stroke [24]. In the present study, we further found that C + P and DHC conferred neuroprotection partially through inhibition of cerebral gluconeogenesis evidenced by PCK enzymes.

In conclusion, the present study found that PH was effective in conferring therapy to rats undergoing AIS through a pathway involving the prevention of dysfunctional gluconeogenesis and hyperglycolysis. These findings justify the exploration of new therapies that target gluconeogenesis, not only in stroke patients, but also in other forms of brain injuries such as trauma and epilepsy.

## 4. Materials and Methods

### 4.1. Subject

All experimental design and procedures were approved by the Institutional Animal Investigation Committee of Capital Medical University, in accordance with the National Institutes of Health (Bethesda, MD, USA) guidelines for the care and use of laboratory animals. From the 226 adult Sprague Dawley rats (280–300 g, from Vital River Laboratory Animal Technology Co Ltd., Beijing, China) originally enrolled to the study, 208 could be used for experimentation; 3 died after surgery, and 15 were excluded due to unsuccessful surgery. Animals were randomly divided into the following groups: (1) sham-operated group without middle cerebral artery occlusion (MCAO) (*n* = 8 × 2) and (2) 2 h MCAO group followed by 6 h (*n* = 8 × 8), 24 h (*n* = 8 × 8), or 48 h (*n* = 8 × 8) of reperfusion. The MCAO group was further randomly divided into 8 subgroups with different treatments: (1) vehicle with saline, (2) C + P (Sigma-Aldrich, St. Louis, MO, USA); (3) DHC (Sigma-Aldrich, St. Louis, MO, USA); (4) combination of C + P and DHC; (5) phloretin (glucose transporter (GLUT)-1 inhibitor, Sigma-Aldrich, St. Louis, MO, USA); (6) cytochalasin B (GLUT-3 inhibitor, Sigma-Aldrich, St. Louis, MO, USA); (7) TZD (thiazolidinedione, PCK inhibitor, Sigma-Aldrich, St. Louis, MO, USA); and (8) apocynin (nicotinamide adenine dinucleotide phosphate oxidase (NOX) inhibitor, Sigma-Aldrich, St. Louis, MO, USA). After treatment, rats were anesthetized by an intraperitoneal injection of chlorine hydrate (10%, 400 mg/kg), after which, brain tissues were collected immediately for the experiments. At 48 h of reperfusion the infarct volume in animals with MCAO was analyzed, and at 6 or 24 h of reperfusion the protein and biochemical measurements were analyzed. Animals were housed under a 12-h light/dark cycle and were kept in the same animal care facility for the entire duration of the study. All efforts were made to minimize any suffering and to reduce the total number of animals used.

### 4.2. Focal Cerebral Ischemia and Reperfusion

This model has been described previously by us [59]. Briefly, rats were fasted 12 h before surgery and then subjected to two hours of MCAO (right side) using an intraluminal filament. Rats were anesthetized in a chamber with 1–3% isoflurane in a mixture of 70% nitrous oxide and 30% oxygen. Then, they were transferred to a surgical table and anesthesia was maintained with a facemask using 1% isoflurane delivered from a calibrated precision vaporizer. Poly-L-lysine-coated intraluminal nylon (4.0) sutures were used to occlude the MCA, which yielded consistent infarcts and significantly reduced inter-animal variability. Two hours after occlusion, reperfusion was established by withdrawal of the filament. Anesthesia was maintained only during the operation of occluding the MCA and withdrawing of the filament. Rats were awake during MCAO. Buprenorphine 0.01 mg/kg SC was used 30 min before the incision and after surgery every 8–12 h, as needed, for 24 h postoperatively, for analgesia.

### 4.3. Pharmacological Hypothermia with C + P and DHC, and Inhibitor Administration

In ischemia models with 2 h MCAO with reperfusion, 4 mg/kg chlorpromazine and promethazine (C + P, 1:1 ratio), 0.5 mg/kg dihydrocapsaicin (DHC), or a combination of C + P and DHC in 3 mL of saline was injected intraperitoneally at the onset of reperfusion after 2 h ischemia, as previously described by us [17]. In order to maintain and enhance the efficacy of the drugs, a second injection of C + P with 1/3 of the original dose was delivered in 2 h in both C + P and C + P + DHC groups. Alternatively, 100 mg/kg of phloretin (GLUT-1 inhibitor), 0.5 mg/kg of cytochalasin B (GLUT-3 inhibitor), 2.5 mg/kg TZD (PCK inhibitor), or 2.5 mg/kg apocynin (NOX inhibitor) was injected 2 h after the onset of ischemia. Rectal temperature (body temperature) was monitored continuously from before surgery, during hypothermia induction, and until its return to baseline (24 h after reperfusion).

### 4.4. Infarct Volume

Infarct volumes were evaluated at 48 h of reperfusion in all rats. The infarct region, defined as the area with reduced staining, was cut into 2 mm thick slices with a brain matrix and was determined in sets of serially cut sections through the MCA territory including the frontoparietal sensorimotor cortex and the dorsolateral striatum, at six different levels from anterior +1.00 mm to posterior −4.8 mm, to the bregma of the brain. We used our routine 2, 3, 5-triphenyltetrazolium chloride (TTC, Sigma, St. Louis, MO, USA) staining at 37 °C. An indirect method for calculating infarct volume was used to minimize error caused by edema. The infarct volumes were determined by using image analysis software (Image J), and calculated according to the following formula: (area of the contralateral hemisphere–area of the nonischemic region in the ipsilateral hemisphere)/area of the contralateral hemisphere 100% [60].

### 4.5. Neurological Deficits

The severity of neurological deficits were evaluated using the modified scoring systems (5 and 12 scores) proposed by Longa et al. [61] and Belayev et al. [62] before surgery for baseline, after 2 h MCA occlusion (during MCA occlusion, just before reperfusion), and after 48 h reperfusion. Higher scores indicate more severe deficits in both scoring systems. The severity and consistency of brain damage in each group were important to this study. After MCA occlusion, the modified scoring system (five score) for neurological deficits was used to confirm brain injury. The MCA occlusions were considered unsuccessful and the rats excluded from the study if the score was 1 or below; approximately 10% of animals with MCA occlusion were discarded for this reason.

### 4.6. Cerebral Glucose, Lactate, and ATP Production

Glucose and lactate levels in the brain were detected using the glucose and lactate assay kits (BioVision, Exton, PA, USA), as previously described by us [47]. Right cerebral hemispheres, including the frontoparietal cortex and striatum supplied by MCA, were extracted and homogenized, then measured by a DTX-880 multimode detector at an absorbance wavelength of 570 or 450 nm, respectively. ATP levels were determined by an ATP colorimetric/fluorometric assay kit (BioVision, Exton, PA, USA) according to the manufacturer’s protocols, as described previously by us [59]. Briefly, a 50 µL brain sample in ATP assay buffer and 50 µL ATP reaction mix were added to the 96 plate wells and incubated for 30 min at room temperature while avoiding any exposure to light. Absorbance of 570 nm was then detected.

### 4.7. ROS Production

ROS was measured by assessing H_2_O_2_ with hydrogen peroxidase linked to a fluorescent compound, as described previously by us [63]. Briefly, homogenized brain samples taken from the MCA supplied territory (cortex and striatum) were diluted to 10 mg/mL. H_2_O_2_ levels in brain homogenates were determined at 37 °C on a DTX-880 multimode detector.

### 4.8. NADPH Oxidase (NOX) Activity

Brain samples of the MCA supplied regions were homogenized in PBS buffer (120 mM NaCl, 4.8 mM KCl, 1.2 mM MgSO_4_, 2.2 mM CaCl_2_, 0.15 mM Na_2_HPO_4_, 0.4 mM KH_2_PO_4_, 20 mM HEPES, 5 mM NaHCO_3_, and 5.5 mM glucose) and a protease inhibitor cocktail. An 80 µL homogenizing buffer supplemented with 6.25 µM lucigenin mix and 20 µL of homogenate were added to each well of the luminescence plate and incubated for 10 min at 37 °C. The reaction was initiated after adding NADPH (100 µM) and luminescence was recorded with a DTX-880 multimode detector every 30 s for a total of 5 min [47].

### 4.9. Cell Death

The level of apoptotic cell death was measured using a commercial enzyme immunoassay kit determining cytoplasmic histone-associated DNA fragments (Roche Diagnostics, San Francisco, CA, USA), as described previously by us [20]. Absorbance of 405 nm was detected with a multimode detector (Beckman DTX-880, Indianapolis, IN, USA).

### 4.10. Protein Expression

Western blot analysis was used to assess protein expression in the ischemic tissue, as described previously by us [42]. Briefly, proteins were extracted from rat brain isolates and loaded onto gels for electrophoresis. Then, the proteins were transferred to a polyvinylidene fluoride membrane. Membranes were incubated with a primary antibody overnight at 4 °C. Primary antibodies included anti-PFK (1:1000, Santa Cruz, CA, USA), anti-GLUT-1 (1:1000, Abcam, Cambridge, MA, USA), anti-GLUT-3 (1:1000, Santa Cruz, CA, USA), anti-LDH (1:1000, Santa Cruz, CA, USA), anti-PCK1 (1:1000, Cell Signaling Technology, Danvers, MA, USA), anti-PCK2 (1:1000, Cell Signaling Technology, Danvers, MA, USA), anti-gp91 (1:1000, Abcam, Cambridge, MA, USA), and anti-β-actin (1:1000, Santa Cruz, CA, USA). Next, membranes were washed and re-incubated with a secondary antibody (goat anti-rabbit IgG, (Santa Cruz, CA, USA)) for 1 h at room temperature. Target protein expressions were visualized using an enhanced chemiluminescence kit (Millipore, Billerica, MA, USA). Western blot images were analyzed using an image analysis program (Image J 1.42, National Institutes of Health, Bethesda, MD, USA) to quantify protein expression in terms of relative image density. The mean amount of protein expression in the control group was assigned a value of 1 to serve as reference.

### 4.11. Statistical Analysis

The statistical analyses were performed with Graphpad Prism V8.0.2 (Graphpad Software, San Diego, CA, USA). The D’Agostino–Pearson test was used to assess normal distribution. The differences among groups were calculated using a one-way ANOVA (after confirming normal distribution) or Kruskal–Wallis test with a significance level set at *p* < 0.05. Post hoc comparison between groups used the least significant difference method. The data and values are all expressed as the mean ± SD.

## Data Availability

The raw data supporting the conclusions of this article will be made available from the corresponding author upon reasonable request.

## Supplementary Materials

The following supporting information can be downloaded at: https://www.mdpi.com/article/10.3390/biom12060851/s1, Table S1: Physiologic Parameters during Transient MCA Occlusion.
