# Supplementary material for: Neuroprotective Effects of Pharmacological Hypothermia on Hyperglycolysis and Gluconeogenesis in Rats after Ischemic Stroke
Source: Biomolecules. 2022 Jun 19;12(6):851. doi: 10.3390/biom12060851 (PMC9220898; doi:10.3390/biom12060851)
Supplement: Supplementary file 1 [file biomolecules-12-00851-s001.zip › biomolecules-1619191-supplementary material - Physiologic Parameters .docx]

| **Table 1. Physiologic Parameters during Transient MCA Occlusion** | | | | | | | | |
| --- | --- | --- | --- | --- | --- | --- | --- | --- |
| **Physiologic Parameters** | **Stroke** | **C+P** | **DHC** | **C+P+DHC** | **Phloretin** | **Cytochalasin B** | **TZD** | **Apocynin** |
| **MAP** |  |  |  |  |  |  |  |  |
| Pre MCAO | 88.9±2.9 | 86.4±2.8 | 83.3±4.2 | 83.9±1.9 | 84.5±2.5 | 87.3±2.9 | 88.0±3.2 | 86.8±2.2 |
| Pre reperfusion | 88.0±2.3 | 86.6±2.3 | 87.0±2.6 | 87.1±1.6 | 85.6±2.3 | 86.2±2.3 | 85.1±2.3 | 86.2±2.3 |
| 2h after reperfusion | 80.2±3.1 | 86.8±2.7 | 83.8±3.5 | 82.2±2.8 | 83.8±2.8 | 84.8±2.7 | 83.2±3.3 | 85.8±3.4 |
| **PH** |  |  |  |  |  |  |  |  |
| Pre MCAO | 7.41±0.02 | 7.39±0.02 | 7.38±0.01 | 7.42±0.02 | 7.40±0.02 | 7.43±0.03 | 7.40±0.02 | 7.39±0.02 |
| Pre reperfusion | 7.36±0.02 | 7.39±0.02 | 7.38±0.03 | 7.39±0.03 | 7.39±0.02 | 7.38±0.03 | 7.37±0.02 | 7.38±0.02 |
| 2h after reperfusion | 7.39±0.03 | 7.35±0.03 | 7.40±0.02 | 7.38±0.03 | 7.36±0.03 | 7.37±0.02 | 7.38±0.03 | 7.36±0.03 |
| **PO_2_** |  |  |  |  |  |  |  |  |
| Pre MCAO | 130.9±6.4 | 138.9±5.6 | 134.2±4.9 | 138.5±6.1 | 140.9±7.1 | 132.9±7.1 | 140.8±6.4 | 141.9±7.1 |
| Pre reperfusion | 133.9±4.8 | 134.6±5.8 | 135.9±4.0 | 130.9±4.3 | 135.6±3.8 | 137.6±4.6 | 136.4±2.9 | 138.6±5.2 |
| 2h after reperfusion | 141.1±6.9 | 134.7±4.7 | 130.1±3.5 | 133.1±6.7 | 135.7±4.2 | 135.1±5.7 | 129.1±4.6 | 135.9±6.7 |
| **PCO_2_** |  |  |  |  |  |  |  |  |
| Pre MCAO | 45.5±2.1 | 43.9±2.2 | 48.0±4.1 | 47.5±3.1 | 45.5±2.2 | 46.3±4.1 | 45.9±2.2 | 44.8±3.5 |
| Pre reperfusion | 42.8±1.5 | 46.7±1.9 | 42.4±2.6 | 46.8±3.4 | 44.7±1.9 | 43.4±2.6 | 45.8±1.5 | 45.7±2.3 |
| 2h after reperfusion | 49.3±2.3 | 46.6±4.5 | 45.2±2.3 | 49.5±2.7 | 47.6±3.5 | 46.0±2.2 | 47.3±3.3 | 45.8±3.5 |
